# Efficacy of Anakinra in Pericarditis: A Systematic Review

**DOI:** 10.7759/cureus.29862

**Published:** 2022-10-03

**Authors:** Ameer Haider Cheema, Keyur Chaludiya, Maham Khalid, Marcellina Nwosu, Srujana Konka, Walter Y Agyeman, Aakash Bisht, Ankit Gopinath, Ana P Arcia Franchini

**Affiliations:** 1 Internal Medicine, California Institute of Behavioral Neurosciences & Psychology, Fairfield, USA; 2 Research, California Institute of Behavioral Neurosciences & Psychology, Fairfield, USA

**Keywords:** anti-interleukin-1 treatment, interleukin-1 inhibitor, treatment resistant pericarditis, idiopathic pericarditis, recurrent pericarditis, anakinra

## Abstract

Inflammation of the pericardium is referred to as pericarditis, which can cause sharp chest pain and has a high chance of recurrence even after treatment. This review will explore anakinra, which is an interleukin-1 receptor antagonist, as a potential new treatment for pericarditis. The systematic review was conducted following the Preferred Reporting Items for Systematic Reviews and Meta-Analyses (PRISMA) guidelines by searching PubMed and GoogleScholar from the years 2012 to 2022. After applying inclusion and exclusion criteria, thorough screening, and quality appraisal, a total of eleven studies were included in the review; eight case reports and three clinical trials. All studies showed that 100 mg/day of anakinra caused a remarkable improvement in patient outcomes. In addition, the pericarditis resolved quicker and had a lower chance of recurrence in comparison to conventional therapy.

## Introduction and background

The pericardium is a double-layer membrane that surrounds the heart. Thickening and inflammation of this membrane is called pericarditis, which accounts for 5% of the chest pain presentations to the emergency department [1]. The recurrence rate is up to 24% even with treatment [1]. The most common cause of pericarditis is idiopathic and is presumed to be caused by viruses such as coxsackie A and B, echovirus, adenovirus, human immunodeficiency virus (HIV), herpes virus, Epstein-Barr virus (EBV), Cytomegalovirus (CMV), influenza, and parvovirus B19 [2].

Other causes include acute myocardial infarction, renal failure, chest trauma, postpericardiotomy, radiation therapy, malignancy, and cardiac procedures as well as autoimmune inflammatory diseases like hypothyroidism, Wegner’s granulomatosis, systemic lupus erythematosus, scleroderma, inflammatory bowel disease, and rheumatoid arthritis to name a few [1,2]. Medications such as doxorubicin, isoniazid, phenytoin, and procainamide are also known to cause inflammation of the pericardium. However, unlike other causes, pericarditis that arises due to medications usually resolves with the discontinuation of the offending agent [1]. 

The above list is not exhaustive, and a diagnostic workup should be done when clinical suspicion is high. Patients commonly present with fever, malaise, and myalgias. There is usually a sharp, pleuritic chest pain that may radiate to the trapezius, neck, arms, or jaw. The pain is generally relieved by leaning forward and aggravated by lying flat [1]. The classic finding on auscultation is a triphasic friction rub heard best along the left sternal border, however, friction rubs are often evanescent and may vary in quality. Inflammatory markers such as erythrocyte sedimentation rate (ESR), C-reactive protein (CRP), and leukocyte count are usually elevated [1].

Diagnosis

According to the European Society of Cardiology 2015 guidelines, the diagnosis of pericarditis is confirmed when two of the following four findings are present [2]: pericardial chest pain, classic auscultation sound of a triphasic scratchy friction rub, PR depression or diffuse ST elevation on electrocardiogram (ECG), and pericardial effusion on imaging such as echocardiography, CT, or MRI.

Treatment

Conventional treatment of pericarditis consists of a mixture of steroids, colchicine, and nonsteroidal anti-inflammatory drugs (NSAIDs) such as aspirin and ibuprofen [1,2]. Pericarditis complicated by tamponade can be treated with pericardiocentesis or the creation of a pleuropericardial window [2]. Approximately one in four patients will have a recurrence after treatment within the first few weeks, which is a significant concern with the current therapy [1,2]. Several medicines are currently under study to enhance the currently available treatment regimens, including the monoclonal antibodies anakinra, canakinumab, and rilonacept [3].

Concerns with current treatments

NSAIDs

No randomized control trial to date has proven the efficacy of NSAIDs in treating acute pericarditis [4]. Recommendations are based on clinical experience alone [4]. Additionally, NSAIDs have been documented to increase the risk of gastrointestinal ulcers by 3.8 times [5]. Other harmful effects include kidney failure, arterial hypertension, and bleeding [4].

Colchicine

Colchicine in combination with an NSAID has proven to be a better option than NSAID monotherapy. However, various studies have shown that the recurrence rate of pericarditis is still unacceptably high (Table 1). Moreover, colchicine therapy in concomitant myocarditis is controversial. In mouse trials, colchicine showed increased mortality when given to mice with myocarditis and pericarditis [6]. Colchicine is also known to cause gastrointestinal disturbances in 5-8% of patients, which is severe enough to warrant discontinuation of therapy [4]. Other side effects include neuromuscular toxicity, aplastic anemia, and myelosuppression [4]. 

**Table 1:** Recurrence rate in various trials using colchicine in combination with non-steroidal anti-inflammatory drugs (NSAIDs)

Trial	Abbreviation	Year	Patients	Recurrence %
Colchicine for Acute Pericarditis [7]	COPE	2005	120	11.7
Colchicine for Recurrent Pericarditis [8]	CORE	2005	84	24
Colchicine for Recurrent Pericarditis [9]	CORP	2011	120	24
Investigation on Colchicine for Acute Pericarditis [10]	ICAP	2013	240	16.7
Efficacy and Safety of Colchicine for Treatment of Multiple Recurrences of Pericarditis [11]	CORP-2	2014	240	21.6
Colchicine Administered in the First Episode of Acute Idiopathic Pericarditis [12]	CAFE-AIP	2019	110	7.8

Corticosteroids

Steroids are considered secondary or tertiary treatments for pericarditis. Interestingly, steroids have led to a prolonged disease course and a higher risk of recurrence [4]. The Colchicine for Acute Pericarditis (COPE) trial showed steroid usage caused a 4.3 times increased risk of recurrence [7]. A meta-analysis by Lotrionte et al. [13] further proved this association which showed that low-dose steroids caused fewer recurrences than high-dose steroids. Additionally, prolonged usage of corticosteroids is well documented in the literature to cause various adverse effects.

Anakinra

**Figure 1:**
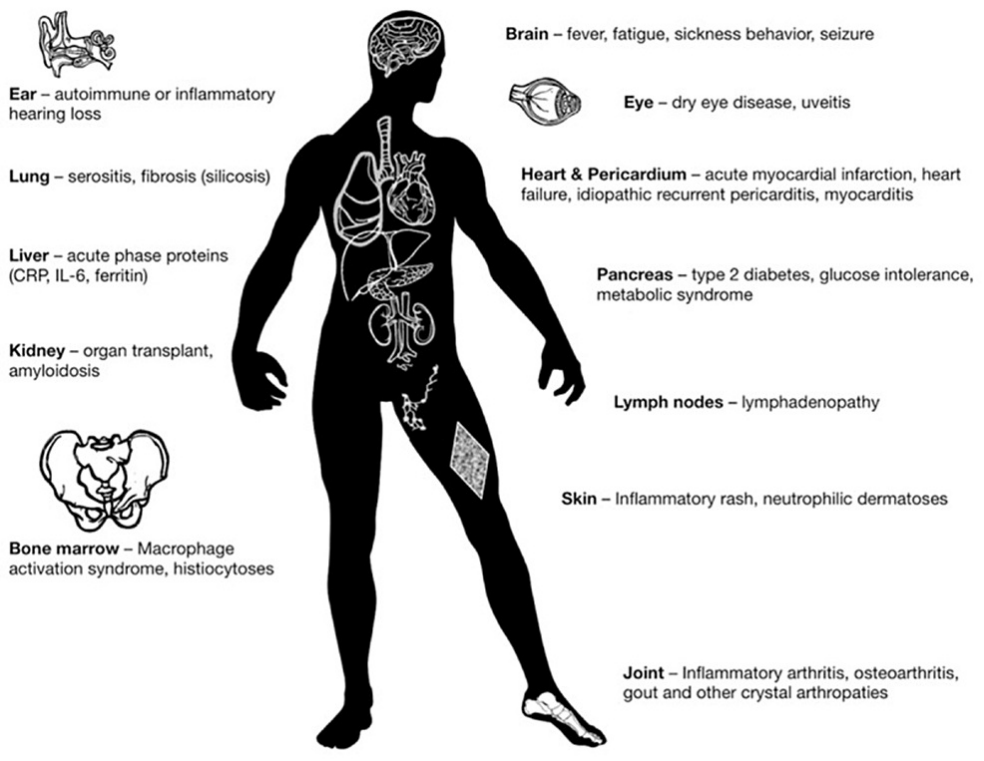
Anakinra's potential uses Image source: Cavalli and Dinarello, 2018 [14]; Reprinted with permission.

Due to the lack of an optimal treatment for pericarditis, and the high risk of recurrence posttreatment, this systematic review aims to discuss a new and emerging treatment for pericarditis: anakinra. Anakinra is a recombinant human interleukin-1 (IL-1) receptor antagonist that blocks both IL-1 alpha and IL-1 beta to prevent inflammation; therefore, it can potentially be used to treat a multitude of inflammatory diseases, as shown in Figure 1 [14]. This review will discuss the appropriateness of anakinra concerning pericarditis. Various clinical studies and case reports that have attempted to treat pericarditis using anakinra will be discussed below. This new and emerging treatment might be more efficacious, safer, and quicker compared to conventional therapy.

Methods

We followed the Preferred Reporting Item for Systematic Reviews and Meta-Analysis (PRISMA) guidelines 2020 [15]. A comprehensive literature search was done using two electronic databases, PubMed and Google Scholar, on July 20th, 2022.

Inclusion and exclusion criteria

We included case reports, clinical trials, and observational studies from 2012 to 2022. Males and females of all ages were included. Only peer-reviewed free full-text articles were selected. Gray literature and non-English language articles were excluded.

Search strategy

PubMed Search

A search was made using the keywords and Boolean “Anakinra AND Pericarditis”. Filters were applied to only show free full-text articles in the English language from 2012 to 2022. Additional filters were used to show clinical studies and case reports exclusively. An attempt was also made using PubMed’s Medical Subject Headings (MeSH) tool. 

Google Scholar Search

A search was made using the keywords and Boolean “Anakinra AND Pericarditis”. A filter was applied to only show studies which included these keywords in the title. Table 2 shows the keyword search terms and MeSH search results. 

**Table 2:** Keyword Search

Keywords	Database	Studies
Anakinra AND Pericarditis	PubMed	137
Anakinra AND Pericarditis	Google Scholar	4900
( "Interleukin 1 Receptor Antagonist Protein/administration and dosage"[Majr] OR "Interleukin 1 Receptor Antagonist Protein/agonists"[Majr] OR "Interleukin 1 Receptor Antagonist Protein/antagonists and inhibitors"[Majr] OR "Interleukin 1 Receptor Antagonist Protein/drug effects"[Majr] OR "Interleukin 1 Receptor Antagonist Protein/immunology"[Majr] OR "Interleukin 1 Receptor Antagonist Protein/organization and administration"[Majr] OR "Interleukin 1 Receptor Antagonist Protein/pharmacokinetics"[Majr] OR "Interleukin 1 Receptor Antagonist Protein/pharmacology"[Majr] OR "Interleukin 1 Receptor Antagonist Protein/physiology"[Majr] OR "Interleukin 1 Receptor Antagonist Protein/therapeutic use"[Majr] OR "Interleukin 1 Receptor Antagonist Protein/toxicity"[Majr] ) AND ( "Pericarditis/chemically induced"[Majr] OR "Pericarditis/complications"[Majr] OR "Pericarditis/diet therapy"[Majr] OR "Pericarditis/drug therapy"[Majr] OR "Pericarditis/immunology"[Majr] OR "Pericarditis/microbiology"[Majr] OR "Pericarditis/prevention and control"[Majr] OR "Pericarditis/rehabilitation"[Majr] OR "Pericarditis/surgery"[Majr] OR "Pericarditis/therapy"[Majr] )	PubMed	41 Full-free text:10

Results

PubMed

The original PubMed search yielded 137 results. After the application of filters and the inclusion and exclusion criteria, there were 15 remaining articles. These articles were then screened based on title, abstract, and full text for relevancy, leading to the removal of five more articles due to irrelevancy. The remaining 10 studies were then critically appraised with standardized quality assessment tools. The attempted search using MeSH did not result in any unique studies.

Google Scholar

**Figure 2:**
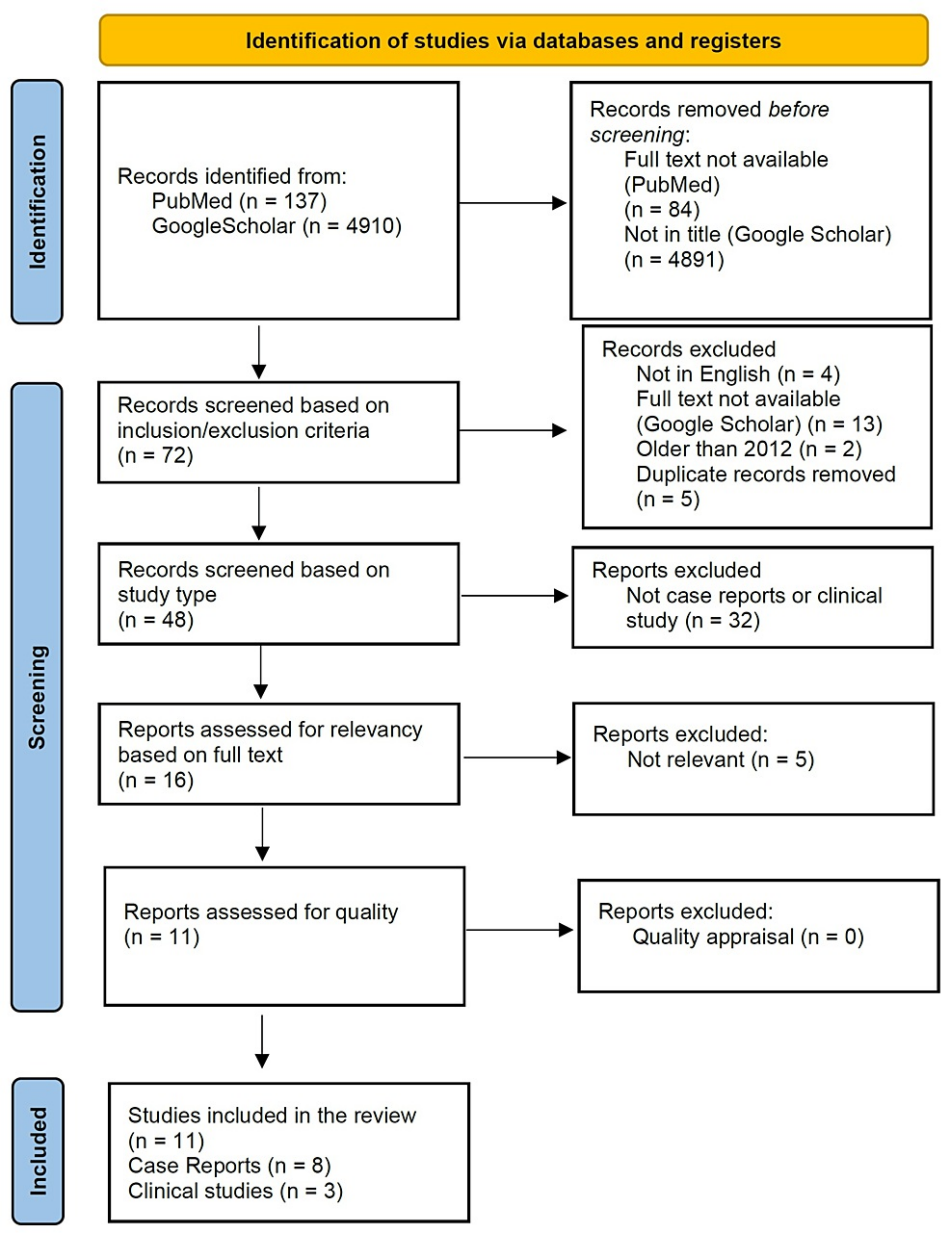
Preferred Reporting Item for Systematic Reviews and Meta-Analysis (PRISMA) flow diagram

The original search yielded 4900 results and after the application of the first filter, 4881 studies were removed. Of the 19 remaining studies, 13 were inaccessible, and five were duplicates, leaving only one study available for quality appraisal. The PRISMA flow diagram for identifying studies is presented in Figure 2.

After studies were identified using our search strategy, applying the inclusion and exclusion criteria and screening the articles for relevancy, eight case reports, two prospective trials, and one retrospective clinical study remained. All studies passed the quality appraisal. The summary of clinical studies is given in Table 3.

**Table 3:** Summary of clinical studies AIRTRIP=Anakinra-Treatment of Recurrent Pericarditis; IRAP=International Registry of Anakinra for Pericarditis

Name	Location	Study Type	Number of Patients	Quality Appraisal Tool
Brucato et al. [16] 2016 Anakinra-Treatment of Recurrent Pericarditis (AIRTRIP)	Italy	Randomized controlled trial	21	Cochrane risk bias assessment tool [17]
Imazio et al. [18] 2020 International Registry of Anakinra for Pericarditis (IRAP)	Canada, Israel, Italy, Slovenia, United States of America	Non-randomized controlled trial	224	Newcastle-Ottawa Quality Assessment [19,20]
Shaukat et al. [21] 2020	United States of America	Retrospective study	34	Newcastle-Ottawa Quality Assessment [19,20]

The case reports were assessed for quality using the Joanna Briggs Institute (JBI) check tool [22]. A minimum requirement of six out of eight (75%) was set. All studies passed the minimum requirement. The assessment of the case reports is summarized in Table 4.

**Table 4:** Critical assessment of case reports

Question	Ahmed et al., 2021 [23]	Karadeniz et al., 2020 [24]	Ocon et al., 2018 [25]	Perna et al., 2022 [26]	Shaukat et al., 2019 [27]	Signa et al., 2020 [28]	Thallapally et al., 2021 [29]	Tomelleri et al., 2018 [30]
Patient’s characteristics described?	Yes	Yes	Yes	Yes	Yes	Yes	Yes	Yes
Patient’s History described?	Yes	Yes	Yes	Yes	Yes	Yes	Yes	Yes
Condition on presentation described?	Yes	Yes	Yes	Yes	Yes	Yes	Yes	Yes
Diagnostic methodology described?	Yes	Yes	Yes	Yes	unclear	No	No	Yes
Treatment/ Intervention described?	No	Yes	Yes	Yes	Yes	Yes	Yes	Yes
Follow up Described?	Yes	Yes	Yes	Yes	Yes	Yes	Yes	Yes
Adverse Events documented?	Yes	Yes	Yes	Yes	Yes	Yes	Yes	Yes
Takeaway lessons?	Yes	Yes	Yes	Yes	Yes	Yes	Yes	Yes
Evaluation	7/8	8/8	8/8	8/8	7/8	7/8	7/8	8/8
Included	Yes	Yes	Yes	Yes	Yes	Yes	Yes	Yes

## Review

Clinical Studies

Three clinical studies were included in the review and in every study, anakinra was found to have better outcomes in comparison to placebo or traditional therapy. The individual studies are discussed below. 

The Anakinra-Treatment of Recurrent Pericarditis (AIRTRIP) [16] was a randomized placebo-controlled trial conducted in 2016 that attempted to investigate the efficacy of anakinra in pericarditis. It involved 21 patients with more than three relapses, associated with raised CRP levels, and colchicine resistance. Patients were given 2 mg/kg/day of anakinra with a maximum dose of 100 mg/day for two months, after which, all the patients became asymptomatic. There were then randomized to receive either a placebo or anakinra for the following six months. At a median follow-up of 14 months, nine out of 10 patients relapsed in the placebo group, while only two out of 11 anakinra patients relapsed.

Published in 2020, the International Registry for Pericarditis (IRAP) [18] was a multicenter study of 224 patients with colchicine-resistant pericarditis. They were treated with 100 mg/day of anakinra for six months, followed by a tapering period of three months. Recurrences decreased from 2.33% to 0.39% after six months, and there was a 91% reduction in emergency department visits and an 86% reduction in hospitalizations. Steroid use decreased from 80% to 27%, and 135 out of 224 patients were stable enough to discontinue anakinra after 18 months. There was an association between the length of treatment and a decreased risk of relapse. Anakinra allowed for a reduction in recurrence of pericarditis from a mean of one recurrence every 157 days to a mean of one every 939 days.

Shaukat et al. [21] published a retrospective report in 2020 on pericarditis patients that were intolerant or refractory to conventional therapy consisting of NSAIDs, colchicine, and steroids. Twelve patients received anakinra plus conventional therapy, while 22 received conventional treatment only. All patients in the anakinra group and 16 out of 22 patients in the conventional treatment group reported resolution of symptoms. Anakinra group had a faster response time of 3.75 +/- 1.87 days compared to 5.63 +/- 3.28 days for the conventional group, and nine conventional therapy patients relapsed during treatment. The authors stated that post-therapy relapse risk was not adequately determined because of inconsistencies in medication delivery. The clinical studies are summarized in Table 5. 

**Table 5:** Summary of prospective and retrospective clinical studies AIRTRIP - Anakinra-Treatment of Recurrent Pericarditis, IRAP - International Registry of Anakinra for Pericarditis

Study	Year	Patient size	Drug Regimen	Result
Imazio et al. [18] (IRAP)	2020	224	100 mg/day	91% reduction in Emergency Department visits and 86% reduction in hospitalizations after six months on anakinra compared to prior treatment
Shaukat et al. [21]	2020	34	100 mg/day	Anakinra group had a higher rate of resolution of symptoms and in a shorter period of time in comparison to the traditional therapy group.
Brucato et al. [16] (AIRTRIP)	2016	21	2 mg/kg/day Max:100 mg/day	Recurrence of pericarditis was significantly lower in the anakinra group compared to the placebo group.

Case Reports

There were nine patients in the case reports studied and remarkably every single patient that was treated with anakinra had better outcomes in comparison to their original regimen. The individual case reports are discussed below.

In 2022, Perna et al. [26] described an acute pericarditis case that appeared after the patient received his second dose of the Pfizer-BioNTech messenger ribonucleic acid (mRNA) vaccine. Pericardiocentesis was done to drain the pericardial effusion, and the patient was started on 600 mg of ibuprofen thrice daily. The patient improved until tapering of ibuprofen was attempted, which led to the recurrence of pleuritic chest pain and fever. Echocardiography at this point showed a large pericardial effusion. Pericardiocentesis was planned again; however, that was deferred in favor of trying anakinra. 100 mg twice on the first day and once per day afterward. The patient's chest pain and fever resolved within a few hours of administration. In 72 hours, the pericardial effusion had resolved entirely and the inflammatory markers had normalized. The patient was discharged on anakinra, ibuprofen, and a proton pump inhibitor and remained asymptomatic on his one-month follow-up.

Ahmed et al. [22] described a 44-year-old female with granulomatosis with polyangiitis (GPA) (Wegener's granulomatosis) complicated by pericarditis. She was diagnosed with GPA via renal biopsy and was initially treated with prednisone and rituximab; however, the patient progressed to end-stage renal disease. During a dialysis session, she presented with pleuritic chest pain, shortness of breath, and fever and was found to have a small pericardial effusion on a transthoracic echocardiogram (TTE). She was initially started on prednisone and colchicine; however, the patient relapsed whenever a steroid taper was tried. This continued for a period of 1.5 years. Eventually, anakinra was tried and this resulted in the improvement of her pericarditis symptoms. Another 1.5 years later, the patient was asymptomatic, and a decision was made to taper off anakinra within a year.

Thallapally et al. [29] described a 44-year-old male that complained of shortness of breath and chest tightness after recovery from an upper respiratory tract infection. Heart sounds were unremarkable, but a CT scan and an echocardiogram showed a small pericardial effusion. The patient was started on naproxen 500 mg twice daily and colchicine 0.6 mg once daily. However, the symptoms progressively worsened, and the patient required methylprednisone in the emergency department. An echocardiogram was repeated, which revealed that the effusion had enlarged. The patient was started on a mixed regimen of ibuprofen, colchicine, and prednisone; however, there were exacerbations of her symptoms whenever a dose reduction was attempted. Additionally, the patient suffered from side effects of prolonged steroid dosing such as headaches, weight gain, facial pressure, and blurry vision. The patient was subsequently started on anakinra 100 mg/day, and a marked improvement in symptoms and laboratory results were seen in two weeks. The patient was continued on anakinra for four months but had a slight elevation in his liver function tests. The patient claimed this was due to his excessive alcohol consumption. Anakinra was discontinued, but the patient did not suffer from any further relapses.

Karadeniz et al. [24] described a 33-year-old male with typical chest pain of pericarditis that was relieved on leaning forward. The patient tested positive for COVID-19 and was initially treated with hydroxychloroquine and moxifloxacin per local recommendations. However, on hospital day three, the chest pain persisted, and echocardiography showed a circumferential pericardial effusion. Initially, the patient was given 0.5 mg colchicine and 25 mg indomethacin. Over the next five days, the patient’s chest pain continued, and his CRP and D-dimer levels rose. At this point, a decision was made to start the patient on 100 mg/day of anakinra. The patient had a remarkable improvement in chest pain, and his CRP and D-dimer returned to baseline in seven days. Anakinra was discontinued, and the patient did not have any relapses after that.

Shaukat et al., 2019 [27], described a case of a 54-year-old woman that had colchicine-intolerant, corticosteroid-dependent recurrent pericarditis. The patient had a history of radiation to the chest for non-Hodgkin’s lymphoma and developed complete heart block that required pacemaker placement. The patient developed urticaria after NSAIDs and diarrhea after colchicine; therefore, both medications were deemed unsuitable. In three months, five pericardiocenteses were done for recurrent pericardial effusions. It was finally decided to treat her with anakinra 100 mg daily. This led to the resolution of symptoms within 72 hours. At her four-month follow-up, the patient was asymptomatic, and no evidence of pleural effusion was seen on chest X-ray.

Ocon et al. [25] described a 61-year-old male with adult-onset Still’s disease (AOSD). The patient presented eight weeks after an initial episode of Lyme disease with spiking fevers, night sweats, generalized malaise, chest pressure, and a dry cough. The patient fulfilled five of the AOSD criteria, and echocardiography showed a large circumferential pericardial effusion. Initially, the patient underwent pericardiocentesis which drained 1400 mL of hemorrhagic fluid. The pericardial drainage tube continued to drain approximately 30 mL of fluid daily, and the patient had malaise and arthralgia along with a fever of 39.2°C. The patient was started on methylprednisone 60 mg IV every 12 hours and anakinra 100 mg daily. Drainage from the pericardiotomy tube decreased to 12 mL after a day and nearly 0 mL after 48 hours. His fever resolved, and his generalized malaise also improved. Repeat imaging with TTE showed an improvement in pericardial effusion, and lab values were moving towards normal. At two weeks and a second follow-up at three months, the patient remained symptom-free while taking anakinra. 

Tomelleri et al. [30] described a 62-year-old male admitted to the Emergency Department (ED) for dyspnea and chest pain. The patient self-reported being diagnosed with idiopathic recurrent pericarditis in the past. He was taking colchicine and NSAIDs with multiple flares that required hospitalization. Due to the severity of the disease, a pleuropericardial window was created. In the next few days, the pleuropericardial window closed spontaneously, and there was a relapse of pericarditis. The patient underwent abdominal CT, which showed a “coated aorta” and “hairy kidney” pointing to a diagnosis of Erdheim-Chester Disease (ECD). The diagnosis of ECD was confirmed on a perinephric biopsy. According to the author, the most commonly prescribed medication for ECD is interferon-alpha; however, it displays limited efficacy. Another option was vemurafenib, which requires months of treatment. It was decided to start the patient on anakinra 100 mg/day. Within two days of treatment, the patient showed a remarkable improvement in clinical symptoms and laboratory values. In the following two months, the patient became completely asymptomatic. Anakinra was continued for another year with no further relapses or complications.

Signa et al. [28] reported a 10-year-old girl that developed recurrent pericarditis after surgical correction of an atrial septal defect. The patient was started on NSAIDs, colchicine, and steroids; however, the girl relapsed once the steroids were stopped and required pericardiocentesis. Five unsuccessful attempts were made at steroid tapering, all leading to relapse. Anakinra 2 mg/kg/day was started leading to a complete resolution of symptoms within a few days. The patient, however, developed an urticarial rash, swelling, and erythema at the injection site, and as such, anakinra had to be discontinued. She was then trialed on canakinumab with steroids and NSAIDs, but she relapsed all four times a steroid taper was attempted. It was decided that the patient should be restarted on anakinra after a desensitizing process with the help of antihistamines and steroids. After successful desensitization, the patient continued receiving daily anakinra for 24 months without relapse. 

In another case report, Signa et al. [28] described an 11-year-old girl with idiopathic recurrent pericarditis. She required a pericardiocentesis at initial diagnosis and benefitted from NSAIDs and colchicine. However, the patient relapsed after ten days, at which point anakinra was started. This led to remarkable improvement with the complete resolution of symptoms. Two months later, the patient was switched to canakinumab due to difficulty in compliance with the daily dosing regimen of anakinra. Within ten days of initiation of canakinumab, the patient relapsed again and required steroid tapering. She was subsequently started on 2 mg/kg/day of anakinra with no relapses at the 26-month follow-up. The case reports are summarized in Table 6. 

**Table 6:** Summary of case reports USA=United States of America; NSAID=non-steroidal anti-inflammatory drug

Author	Year	Country	Age/ gender	Anakinra Regimen	Summary
Perna et al. [26]	2022	Italy	30 M	200 mg once then 100mg/ day	Relapse on ibuprofen treatment. Anakinra provided relief in a few hours.
Ahmed et al. [23]	2021	USA	44 F	unknown	Recurrence after colchicine and prednisone. Anakinra treatment proved successful.
Thallapally et al. [29]	2021	USA	44 M	100 mg/day	Relapsed multiple times on steroid, colchicine, and NSAID therapy. Successfully treated with 4 months of anakinra therapy.
Karadeniz et al. [24]	2020	Turkey	33 M	100 mg/day	Initially tried on indomethacin and colchicine without improvement. Anakinra proved successful.
Shaukat et al. [27]	2019	USA	54 F	100 mg/day	Resolution of symptoms in 72 hours with anakinra
Ocon et al. [25]	2018	USA	61 M	100 mg/day	Improvement after an anakinra and methylprednisone regimen.
Tomelleri et al. [30]	2018	Italy	62 M	100 mg/day	Usage of anakinra resulted in rapid resolution of symptoms.
Signa et al. [28] patient 2	2017	Italy	11 F	2 mg/kg/day	Multiple relapses with all first-line medications until successful treatment with anakinra.
Signa et al. [28] patient 1	2016	Italy	10 F	2 mg/kg/day	Relapsed after all first-line medications. Anakinra provided relief.

As shown above, in the limited case studies and clinical trials, anakinra has proven to be highly effective in reducing symptoms and recurrence. Anakinra has demonstrated the potential to radically change current treatment guidelines. In 2021, a randomized controlled trial comparing anakinra with placebo was terminated early because significant benefits were seen within 24 hours. The researchers deemed it unethical and unnecessary to complete the planned enrollment [31].

Effective dosage regimen

Anakinra is typically administered 100 mg once per day subcutaneously and has a half-life of approximately 2.6 hours [32]. The bioavailability of anakinra is not significantly affected by body mass; therefore, it usually does not require dose adjustments [33]. The only concern is in renal failure patients, where the half-life increases to 7.15 hours, and most authors suggest an alternate form of treatment [32,34]. Previously, the biggest reason for hesitancy in using anakinra was the uncertain nature of the duration of treatment [18]. However, the IRAP study has presented promising evidence that a 100 mg/day treatment for three months followed by tapering for three months significantly reduces the risk of recurrence at 36 months [18].

Drawbacks of anakinra therapy

The major potential disadvantages include a long duration of therapy and a higher cost associated with anakinra [16]. Anakinra reportedly has high incidences of local injection site reactions (38-44%) [18,35]; however, these can be treated with antihistamines or topical corticosteroids [36]. Some authors recommend warming the syringe to room temperature and using a cold pack at the injection site two to three minutes before injecting [36]. In the IRAP study of 224 patients, adverse effects observed include myalgias and arthralgias (6%), elevated transaminases (3%), and neutropenia (1%). Six soft tissue and respiratory infections were observed (3%), and seven patients (3%) had to discontinue anakinra because of adverse effects [18].

Limitations

The search strategy for the review was limited to two databases, and only free full-text articles were included, possibly leading to some studies being omitted. Another constraint was that only English language studies were included. A significant data gap could exist as only one case report was about children, and the three clinical trials studied focused on adult patients. While it seems anakinra is equally effective in adult and pediatric patients, more information must be collected before the drug's safety can be confirmed in pediatric patients. Lastly, this review did not include other IL-1 receptor blockers such as rilonacept and canakinumab as they have also shown variable results in small trials and case reports [37].

Suggestions

Anakinra and IL-1 receptor blockers must not become the end-all-be-all treatment as studies should also focus on other immunosuppressives. Methotrexate is one such example that is highly effective in inflammatory conditions such as rheumatoid arthritis and is yet to be studied for pericarditis.

Additionally, more data must be collected, especially randomized controlled trials with a placebo-controlled group, to firmly establish anakinra’s effectiveness and assess its safety. More clinical studies must also be conducted to refine the optimum dosing regimen and tapering duration. Much needs to be done to fully understand disease mechanisms and find solutions, as the current treatment with traditional therapy is inadequate.

## Conclusions

Pericarditis is a challenging disease with high morbidity and frequent recurrences. The limited data has shown promising results, and only the surface has been scratched concerning the treatment of pericarditis with anakinra. The most significant benefits of anakinra can be summarized by the following five points: efficacy in previously refractory cases, rapid onset of action, lower risk of recurrence, quick withdrawal of steroids, and a better side effect profile in comparison to traditional therapy. In light of the above evidence, physicians should be open to trying anakinra, especially when conventional therapy with NSAIDs, colchicine, and steroids fails or the patient has recurrences of pericarditis. The most extensive clinical study to date recommends that a 100 mg/day dosage should be tried for three months and then tapered off over a period of three months. This review should spark discussions and challenge existing narratives as anakinra has the potential to change current guidelines.
